# Targeting multiple nodes of MLL complexes to improve leukemia therapy

**DOI:** 10.18632/oncotarget.21598

**Published:** 2017-10-07

**Authors:** Caroline Dafflon, Ralph Tiedt, Jürg Schwaller

**Affiliations:** Ralph Tiedt: Novartis Institutes for BioMedical Research, Disease Area Oncology, Basel, Switzerland

**Keywords:** DOT1L, Menin, MLL-rearranged leukemia, epigenetics, MLL complexes

In order to sustain an oncogenic state, cancer cells often co-opt chromatin regulatory pathways and the general transcription machinery. Epigenetic dysregulation is particularly well studied in acute leukemia in which the mixed lineage leukemia 1 (*MLL*, *MLL1*, *KMT2A*) gene is subject to chromosomal rearrangements, leading to oncogenic MLL-fusion proteins. *MLL* rearrangements are found in over 70% of acute leukemias in infants and 5-10% in adults, and are generally associated with a poor prognosis [1].

**Figure 1:**
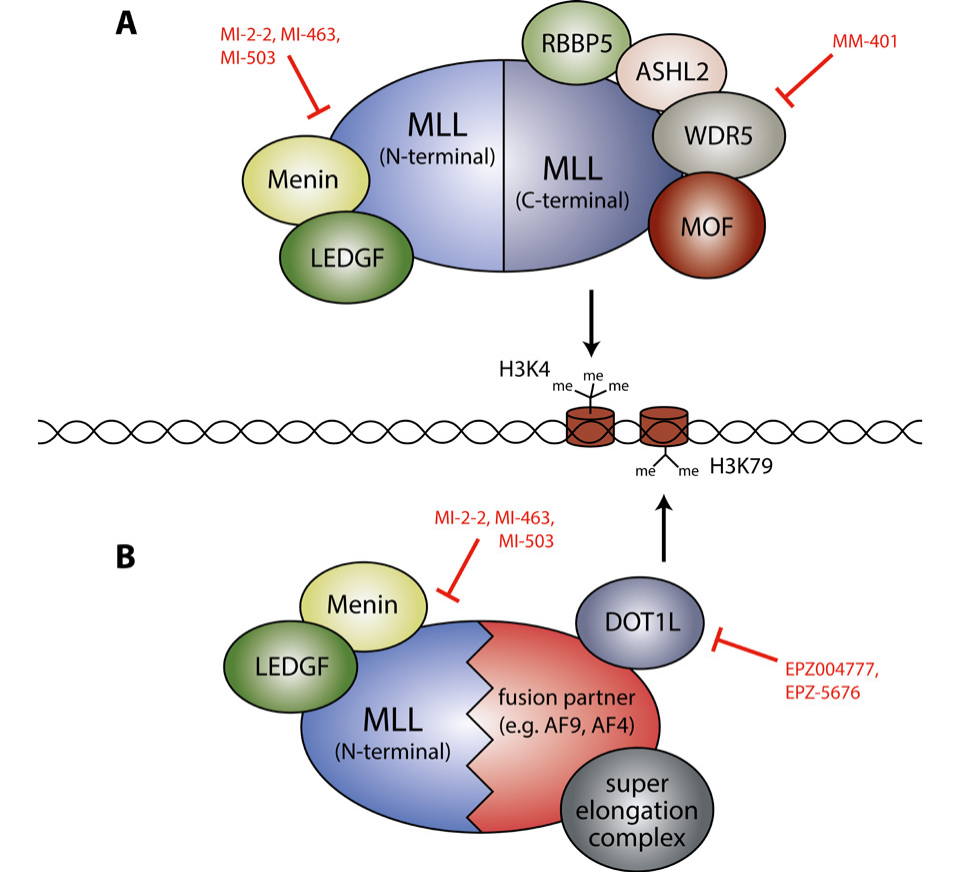
MLL complexes in the context of MLL-rearranged leukemia **A.** Wild-type MLL protein. The N-terminal part is associated with different proteins such as LEDGF and Menin. The C-terminal part interacts with RBBP5, ASH2L, WDR5 and MOF. The SET domain of the MLL C-terminal part catalyzes methylation of H3K4. **B.** MLL-fusion protein. The MLL N-terminal portion is fused to different partners, which are able to recruit co-factors such as DOT1L. Deposition of the H3K79me2 mark by DOT1L is critical for maintaining high expression levels of MLL-fusion target genes. Some potential points for pharmacological intervention and examples of corresponding inhibitors are indicated by red blunt arrows.

The *MLL* gene encodes a large multi-domain methyltransferase with critical functions in embryonic development and hematopoiesis. The C-terminal portion of the protein contains a SET domain that methylates lysine 4 of histone 3 (H3K4), while complex formation with other proteins such as LEDGF (gene symbol *PSIP1*) and Menin (*MEN1*) mediated by the MLL N-terminus is important for specific recruitment to target genes. *MLL* rearrangements are mostly mono-allelic and result in the replacement of the C-terminal portion including the SET domain by one of a large number of different fusion partners, of which AF4, AF9, AF6, AF10, ELL and ENL are the most prevalent. Gain of function of these MLL-fusion proteins is thought to be due to the recruitment of new cofactors such as DOT1L to gene loci that are governed by the N-terminal DNA-binding portion of MLL. DOT1L is the only known H3K79 methyltransferase, and the H3K79me2 mark is broadly associated with active transcriptional elongation. However, recruitment by MLL-fusion proteins appears to drive particularly high levels of H3K79me2 at a subset of genes (e.g. *HOXA9*, *MEIS1*), thereby maintaining their continued high expression, which is critical for the onset and maintenance of leukemia [2](Figure 1).

Recently developed small molecule inhibitors of DOT1L block global H3K79 methylation, suppress MLL-fusion target genes, selectively reduce growth and induce differentiation of *MLL*-rearranged leukemia cells [2, 3]. However, DOT1L inhibition is inefficient and slow acting in many of these models. This observation prompted us to perform a genetic sensitization screen in human *MLL*-rearranged leukemia cell lines using a deep-coverage shRNA library [4]. We scored for genes whose knockdown would specifically decrease cell viability in presence of a DOT1L inhibitor. Interestingly, *MLL* itself and *LEDGF* were the top hits across 5 different cell lines representing both AML and ALL. Other highly ranked hits were known components of MLL-fusion protein complexes including *AF10* (*MLLT10*) and *MOZ* (*MYST3*), or critical downstream target genes such as the histone demethylase *JMJD1C*. Experiments with single shRNAs validated the sensitization potential of *LEDGF* knockdown and suggested that sensitization by *MLL* knockdown stems at least in part from reducing levels of the wild type protein. Collectively, these results indicate that simultaneous targeting of multiple nodes in MLL complexes is able to boost the anti-leukemic effect of DOT1L inhibition.

Menin together with LEDGF is a critical co-factor for DNA-binding of the MLL N terminus. While targeting of LEDGF seems to be challenging, disruption of the interaction between MLL and Menin has been achieved by selective small molecules that substantially reduce the growth of *MLL*-rearranged xenograft models in mice [5]. Menin shRNAs were not part of our screening library, but we could confirm that knockdown of Menin can also sensitize leukemia cells to DOT1L inhibition. We thus evaluated the combination of a MLL-Menin inhibitor (MI-2-2) and a DOT1L inhibitor (EPZ004777) in depth in several leukemia models bearing *MLL* rearrangements. We consistently observed superior but still specific effects of this combination on viability, concomitant with a more efficient suppression of MLL-fusion target genes. Improved disruption of the MLL-Menin interaction is subject to ongoing drug discovery efforts, but no inhibitor has reached the stage of clinical evaluation yet.

The preclinical literature is rich in proposed drug targets for *MLL*-rearranged leukemia, many of which are related to the function of MLL complexes as transcription factors that maintain a critical gene expression program to prevent differentiation. While a pharmacological approach is speculative for several proposed targets so far, the DOT1L inhibitor EPZ-5676 has already been tested in the clinic. However, in a phase 1 study only a subset (∼30%) of patients with *MLL* rearrangements showed evidence of clinical activity, in many cases insufficient to qualify as objective responses [6]. The fact that MLL and its fusions bind to so many different components to exert their effects on transcription suggests a certain degree of redundancy and potential for adaptation. It is therefore not unexpected that targeting a single auxiliary factor is suboptimal, and simultaneous targeting of different nodes may be required to profoundly block leukemogenic MLL-fusion driven transcription programs (Figure 1). Due to its function as master regulator of differentiation and self-renewal, MLL targeting concepts are not necessarily restricted to *MLL*-rearranged leukemia, but may be more broadly applicable in leukemia with other genetic alterations or even in solid cancers. For instance, DOT1L was recently found to be a valid target in *NPM1* mutant leukemia, and combination with a Menin inhibitor showed enhanced activity, suppression of *HOX* genes expression and induction of differentiation [7].

Lastly, there is increasing evidence that presence of the non-rearranged wild type *MLL* allele and/or the *MLL2* gene is critical for malignant transformation by MLL-fusions. A potential pharmacological strategy to inhibit wild type MLL is to target the binding partner WDR5, which is required for H3K4 methyltransferase activity and for the contribution of wild type MLL to leukemogenesis [1](Figure 1). Strikingly, increased stability of the wild type allele by targeted interference of its degradation mechanism was recently proposed to reduce the leukemogenic activity of MLL-fusions [8]. Despite all these emerging strategies, the only clinically relevant targeted AML therapy so far is based on degradation of the fusion protein PML-RARα that drives acute promyelocytic leukemia. Therefore, the goal remains to identify pharmacologic strategies that reduce the dose of the MLL-fusion protein below a critical level to efficiently release the differentiation block and dissolve the leukemic phenotype.
